# Liposomes for the Treatment of Brain Cancer—A Review

**DOI:** 10.3390/ph16081056

**Published:** 2023-07-25

**Authors:** Richu Raju, Waad H. Abuwatfa, William G. Pitt, Ghaleb A. Husseini

**Affiliations:** 1 Biomedical Engineering Program, College of Engineering, American University of Sharjah, Sharjah P.O. Box 26666, United Arab Emirates; 2 Materials Science and Engineering Ph.D. Program, College of Arts and Sciences, American University of Sharjah, Sharjah P.O. Box. 26666, United Arab Emirates; 3 Department of Chemical and Biological Engineering, College of Engineering, American University of Sharjah, Sharjah P.O. Box 26666, United Arab Emirates; 4 Department of Chemical Engineering, Brigham Young University, Provo, UT 84602, USA; pitt@byu.edu

**Keywords:** liposomes, nanocarriers, brain, targeted drug delivery

## Abstract

Due to their biocompatibility, non-toxicity, and surface-conjugation capabilities, liposomes are effective nanocarriers that can encapsulate chemotherapeutic drugs and facilitate targeted delivery across the blood–brain barrier (BBB). Additionally, strategies have been explored to synthesize liposomes that respond to internal and/or external stimuli to release their payload controllably. Although research into liposomes for brain cancer treatment is still in its infancy, these systems have great potential to fundamentally change the drug delivery landscape. This review paper attempts to consolidate relevant literature regarding the delivery to the brain using nanocarriers, particularly liposomes. The paper first briefly explains conventional treatment modalities for cancer, followed by describing the blood–brain barrier and ways, challenges, and techniques involved in transporting drugs across the BBB. Various nanocarrier systems are introduced, with attention to liposomes, due to their ability to circumvent the challenges imposed by the BBB. Relevant studies involving liposomal systems researched to treat brain tumors are reviewed *in vitro, in vivo*, and clinical studies. Finally, the challenges associated with the use of liposomes to treat brain tumors and how they can be addressed are presented.

## 1. Introduction

Cancer, characterized by the uncontrolled proliferation of cells in the body due to genetic mutations, is currently one of the most prevalent diseases worldwide. Over the past 2700 years, medical research has made significant strides in understanding and treating this disease [1,2]. Generally, cancer cells grow and multiply rapidly, producing descendant cells that carry the mutated gene. While these altered cells resemble the original cells, mutations cause them to multiply at an accelerated rate, a condition known as hyperplasia. Over time, these cells undergo further mutations and develop abnormal shapes, a process called dysplasia [3,4]. Two types of genes contribute to tumor formation: oncogenes and tumor suppressor genes [5]. Changes in proto-oncogenes at the cellular level lead to oncogenesis, where proto-oncogenes transform into oncogenes, causing uncontrolled cell division. Oncogenesis can be triggered by mutations in nucleic acid sequences in DNA, chromosomal rearrangements, and gene amplification [6]. Likewise, when tumor suppressor genes undergo mutations or inhibitory processes that limit or halt their function, uncontrolled cell division occurs, forming tumors [7].

Cancer ranks among the leading causes of global mortality, responsible for nearly 9.93 million deaths in 2020, according to the latest global survey on cancer epidemiology. Brain and central nervous system (CNS) tumors were newly reported in 308,162 patients, while globally reported mortalities reached 251,329 [8]. Brain and CNS cancers encompass malignancies in the brain, cranial nerves, spinal nerves, spinal cord, and meninges [9,10]. Although there are over 130 different types and classifications of brain tumors, it is beyond the scope of this article to cover all of them. Instead, this review focuses on the four most common types of brain cancer: metastatic brain tumors, meningioma, astrocytoma, and glioblastoma. Metastatic brain tumors represent the most prevalent type. Brain metastases occur in 10% to 26% of cancer-related deaths [11]. Another type of brain cancer is meningioma, which accounts for nearly fifty percent of benign brain tumors, typically classified as grade 1 tumors. These tumors develop in the meningeal layers of the brain or spinal cord [12]. Astrocytoma arises from a specific type of glial cells called astrocytes, star-shaped brain cells in the cerebrum. Astrocytes regulate brain activities involved in neurogenesis and synaptogenesis by controlling BBB permeability and maintaining extracellular homeostasis [13]. This form of tumor often does not extend beyond the brain and spinal cord, nor does it damage other organs. Most brain areas and, rarely, the spinal cord are susceptible to developing astrocytoma [14]. Lastly, glioblastomas are highly aggressive and malignant grade IV brain tumors that originate from glial cells. They are often categorized as advanced-grade astrocytomas [15,16]. Traditional treatment options for brain tumors encompass surgery, radiation therapy, and chemotherapy [17]. Such conventional drug delivery systems (DDSs) encompass various forms, such as tablets, suspensions, powders, capsules, and sprays, which can be administered through invasive (intravenous) or non-invasive routes (oral, transdermal, mucosal, or nasal). However, these conventional approaches often fail to completely eliminate tumor cells due to their poor solubility, low bioavailability, short half-life, lack of selectivity, inadequate cell interactions, and large particle sizes. Furthermore, chemotherapeutic drugs face the challenge of bypassing both the BBB and the brain–tumor barrier (BTB). Consequently, there has been a surge of interest in targeted drug delivery systems. To overcome these drawbacks, researchers have extensively studied and developed nanoparticle-based DDSs. These advanced systems enable targeted transportation and delivery of drugs and have garnered significant interest for their potential applications in the localized delivery of chemotherapeutic agents [18]. 

Nanocarriers, including liposomes, micelles, hydrogels, gold nanoparticles, iron oxide nanoparticles, carbon-based nanomaterials (such as carbon nanotubes), polymeric nanoparticles, and dendrimers, have undergone extensive research due to their potential as drug delivery vehicles at the nanoscale. These nanoparticle-based DDSs offer several advantages, such as enhanced drug solubility, improved bioavailability, increased stability, prolonged circulation time, and reduced adverse effects [19]. This paper provides a concise overview of brain cancer therapy and concludes with a comprehensive review of the utilization of liposomes in such therapeutic strategies.

### 1.1. Conventional Treatment Methods

**Figure 1:**
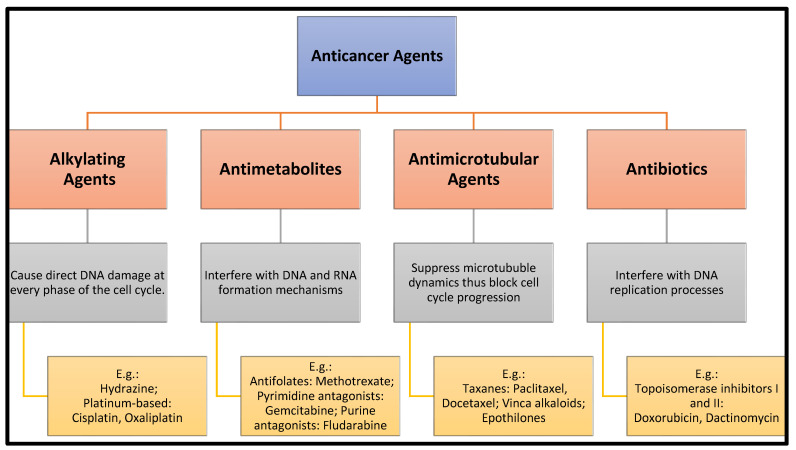
Major classes of chemotherapeutic drugs.

The major classes of chemotherapeutics are outlined in Figure 1. However, these conventional approaches often fall short of eliminating tumor cells, and chemotherapeutic drugs face the challenge of bypassing both the BBB and the brain–tumor barrier (BTB). Consequently, there has been a surge of interest in targeted drug delivery systems.

The side effects of cancer chemotherapy continue to cause significant distress for cancer patients. These side effects primarily stem from the drug’s mechanism of action (MOA) on non-targeted cells [20]. For instance, cytotoxic chemotherapy, designed to eliminate cancer cells, often disrupts the expression of DNA and proteins in normal host cells both near and distant from the target tissue. Consequently, a limited therapeutic index exists, which may lead to potentially lethal toxicity. Most chemotherapy medications exert their effects on rapidly replicating cells by damaging DNA or microtubules. Consequently, unless localized, these drugs affect rapidly replicating cells throughout the body, such as those in the bone marrow, gastrointestinal system, and hair follicles [21]. The most commonly reported side effects are anemia, fatigue, appetite loss, concurrent gastric and digestive issues, myelosuppression, mucositis, alopecia, sterility and infertility, immunosuppression, and peripheral neuropathy [22]. 

Immunotherapy harnesses the body’s innate immune system to combat cancer cells. Cytokines, protein molecules produced by immune cells, stimulate the immune system to generate more T-cells, B-cells, and interleukins, which aid in identifying and eliminating cancerous cells [23]. The two primary forms of immunotherapy are vaccine treatments and cell-transfer therapy. Cancer vaccines activate the patient’s immune system by delivering tumor antigens, such as entire cells, peptides, or nucleic acids, known as tumor-associated antigens (TAAs) and tumor-specific antigens (TSAs). This type of vaccination can induce cellular immunity and humoral immune response to impede tumor development and destroy tumor cells [24,25].

A study conducted by Platten et al. demonstrated the effectiveness of a cancer vaccine specifically developed for individuals with glioma and enzymatic IDH1 mutations in their tumors. The findings revealed a vaccine-induced immune response in over 90 percent of the patients within a cohort of 33 subjects. Additionally, the study reported a three-year progression-free rate of 0.63 and a death-free rate of 0.84 [26]. Another study by Prins et al. reported the successful administration of an autologous tumor lysate-pulsed dendritic cell (DC) vaccination in a study of twenty-three patients diagnosed with glioblastoma. The median time to progression of the tumor (TTP) was 15.9 months. The median overall survival time (OS) was 31.4 months, calculated from the date of the first surgical diagnosis of glioblastoma. At one, two, and three years after the initial diagnosis, the overall survival rate was 91%, 55%, and 47%, respectively, after the administration of the DC vaccine [27].

Adoptive cell therapy is a technique that changes a person’s cells to effectively attack and eliminate tumor cells [28]. The success of adoptive cell transfer (ACT) treatment for cancer patients relies on the ex vivo production of highly active, tumor-specific lymphocytes and their subsequent delivery to the patient [29]. To ensure optimal outcomes, lymphodepletion is performed before ACT to eliminate T regulatory cells and lymphocytes that compete with the transplanted cells for homeostatic cytokines like interleukin-7 (IL7) and interleukin-15 (IL15). Following a lymphodepleting conditioning regimen, autologous antitumor lymphocyte cultures and high-dose interleukin are administered to patients, leading to lymphocytes trafficking to the tumor site and promoting tumor regression [30]. 

Chimeric antigen receptor (CAR) therapy is a specific form of ACT in which a patient’s T-cells are extracted and genetically modified to express a CAR targeting a tumor antigen. These modified cells are then expanded in vitro and re-infused into the patient. In a study by O’Rourke et al. to treat glioblastoma multiforme (GBM), autologous T cells were modified to target the epidermal growth factor receptor variant III (EGFRvIII) mutation. The treatment resulted in a moderate increase in median overall survival of approximately 8 months, with a significant accumulation of CAR-T cells observed at the tumor site through histopathological analysis [31]. In another study by Brown et al., interleukin-13 receptor α2 (IL13Rα2) CAR T cells were administered to three patients with recurrent GBM. IL-13 plays a role in modulating inflammation and the immune response by interacting with the IL13Rα2 pathway. Two of the three patients demonstrated a robust antitumor response to the IL13Rα2 CAR-T cell treatment. IL-13 plays a crucial role in modulating inflammation and the immune response through its interaction with the IL13Rα2 pathway. Remarkably, two of the three patients who received the IL13Rα2 CAR-T cell treatment exhibited a robust antitumor response [32].

A monoclonal antibody (mAb), sometimes abbreviated as moAb, is utilized in targeted therapy, which involves administering lab-synthesized antibodies that specifically target tumor pathways. The Fab (fragment, antigen-binding) region of a mAb is derived from the amino-terminal ends of the light and heavy chains of an immunoglobulin polypeptide. This region, referred to as the variable (V) domain, makes up unique amino acid sequences that distinguish each type of antibody and determine its affinity for antigen binding. Antibodies, also known as immunoglobulins (Ig), are large, Y-shaped proteins the immune system employs to identify and eliminate harmful microorganisms and viruses [33]. These mAbs selectively bind to antigens on cancer cells, stimulating an immune response against the targeted cancer cells [34]. Furthermore, these mAbs can be modified to deliver toxins, radioisotopes, cytokines, or other active agents or engineered to produce bispecific antibodies that can bind to both the target antigen and a conjugate or effector cell through their Fab regions [35]. 

The antitumor effects of monoclonal antibodies (mAbs) are believed to be mediated through distinct pathways, each playing a significant role in the process. These pathways include surface antigen cross-linking, antibody-dependent cellular cytotoxicity (ADCC), complement-mediated cytotoxicity (CMC), inhibition of essential activation signals for cell development, alteration of the cytokine environment, and promotion of an active antitumor immune response [36]. Tumor antigens such as epidermal growth factor receptor (EGFR), cytotoxic T lymphocyte-associated antigen 4 (CTLA4), CD20, CD30, CD52, erbB2, and vascular endothelial growth factor (VEGF) have been studied for targeted drug delivery to the brain [37]. For instance, bevacizumab (BEV), a monoclonal antibody targeting overexpressed VEGF, showed promising results in a study conducted by Nghiemphu et al. [38]. The study compared 44 patients with recurrent glioblastoma (55 years or older, Karnofsky Performance Status (KPS) < 80) treated with BEV to a group of 44 patients who received no further therapies. The BEV-treated group demonstrated an improvement in progression-free survival (PFS) by 2.4 months and overall survival (OS) by 2.9 months [38].

Another example is nimotuzumab, a humanized EGFR monoclonal antibody. In a phase II study, nimotuzumab was administered to 47 children and adolescents with resistant or recurrent high-grade gliomas, resulting in well-tolerated and encouraging outcomes (partial response: 9 percent; stable disease: 22 percent) [39]. In a separate phase II trial by Ramos et al., the combination of nimotuzumab and radiotherapy was examined in 21 patients with malignant gliomas. The study reported a 17 percent complete response and 21 percent partial response, with a median survival time of 22 months, indicating improved survival rates [40].

### 1.2. Drug Delivery across the BBB

The BBB serves as a crucial barrier that separates circulating blood from neural tissues. It has distinct and protective properties that not only regulate the movement of ions and molecules but also maintain brain homeostasis [41]. Considered the most selective barrier in the human body, the BBB is primarily composed of tightly joined endothelial cells connected by tight junctions (TJs) and adherens junctions (AJs), along with a thin layer of pericytes. These closely interconnected components form a robust structure that significantly restricts the permeability of therapeutic compounds, including antineoplastic drugs. Astrocytic glia, pericytes, microglia, and neuronal processes are closely associated and surround the brain capillaries. This specialization of the endothelium of blood vessels entering the brain during development is crucial [42].

Because the BBB remains intact during the early stages of malignant brain tumors, the enhanced permeability and retention (EPR) effect does not occur. The EPR effect refers to the phenomenon where small nanoparticles and macromolecular drugs accumulate more in tumor tissues compared to normal tissues [43]. Generally, the EPR effect is caused by increased pore size in neovascularization and compromised lymphatic clearance found in malignancies. Rapid angiogenesis induced by tumor cells leads to the development of poorly aligned and defective blood vessels [44]. Due to the permeability of tumor vasculature and impaired lymphatic drainage, nanocarriers with extended circulation times preferentially accumulate in the tumor tissue and remain in the tumor bed. The optimal nanoparticle size to achieve the EPR effect in solid tumors while avoiding clearance by the liver and spleen is typically between 100 and 200 nm [45]. Only nanocarriers conjugated with targeting ligands within this size range can cross the BBB and accumulate in tumors [46]. 

Therefore, the BBB poses the most significant challenge for conventional chemotherapy in the early stages of brain malignancies or after surgery [47]. Moreover, cells’ ATP-binding cassette (ABC) efflux pumps, a class of transporter proteins, actively pump various drugs out of cells, leading to drug resistance and lessening intracellular drug concentrations. Multidrug resistance is attributed primarily to the increased activity of efflux pumps in the cell membrane, particularly P-glycoprotein (Pgp), the most common efflux pump. Additionally, several ABC efflux pumps, such as multidrug resistance proteins 4 (MRP4) and breast cancer resistance protein (BCRP), are expressed on the blood side of the BBB [48].

To overcome this active drug export, many anticancer drugs act as substrates for the ABC efflux pumps, for example, P-gp protein transports vincristine, DOX, and etoposide; MRP4 protein transports 6-mercaptopurine and methotrexate; and BCRP protein transports prazosin and nitrofurantoin. Due to the tight junctions (TJs) and the presence of efflux pumps, practically all large-molecule medications and over 98% of small-molecule potential therapies are unable to penetrate brain tissue through paracellular transport [49]. While the BTB is modified near the tumor growth, it still significantly restricts the transport of most therapeutic agents into brain tumors [50].

Although the BTB is more permeable than the BBB, its variable permeability to small and large molecules, along with its heterogeneous ischemia, contributes to the inefficient accumulation of medications in brain tumors [51]. The BBB remains a significant barrier to the successful clinical treatment of brain tumors. However, there are several mechanisms for drug transport across both the BBB and BTB. The transport modes of nanocarriers targeted at the BBB can be broadly categorized into four groups: transporter-mediated transcytosis (TMT), receptor-mediated endocytosis (RME), receptor-mediated transcytosis (RMT), and adsorptive-mediated transcytosis (AMT).

TMT refers to the movement of a solute across a membrane from a high to low concentration by binding the solute to a protein transporter on one side of the membrane, as observed with glucose or amino acids [52]. The energy provided by ATPs can be utilized to transport molecules against a concentration gradient. Glucose transporters (GLUTs), which play a crucial role in transporting glucose from the blood into the brain, have been a significant area of research for nanocarriers targeted at the BBB [53].

The process of RME plays a vital role in cellular mechanisms by facilitating the uptake of specific molecules from the extracellular environment. This process involves the recognition, attachment, and internalization of specific ligands by cell surface receptors, which enables the selective absorption of proteins, lipids, and hormones essential for cellular activities. The aforementioned process consists of a series of stages, starting with ligand-receptor binding, followed by receptor clustering in clathrin-coated pits and the formation of vesicles through invagination and pinching. This results in the shedding of clathrin coats, leading to the sorting and maturation of early endosomes, acidification for the formation of late endosomes, fusion with lysosomes for degradation, and potential recycling of receptors through endosomal recycling. Receptor-mediated endocytosis is crucial for regulating cell signaling, nutrient uptake, and the removal of extracellular substances, thereby maintaining cellular homeostasis and ensuring optimal cellular function [54].

The process of RMT facilitates the selective absorption of macromolecules, including growth factors, enzymes, and plasma proteins, by endothelial cells through their receptors [55]. The initial step in this process involves the binding of a molecule to one of the numerous receptors clustered in coated pits on the plasma membrane. These invaginations extend into the cytoplasm and, when bound to a ligand, pinch off from the plasma membrane, forming coated vesicles [56]. When the endosome becomes more acidic, the ligand is released from the receptor and crosses the membrane to the other side. So far, RMT has been the most widely employed method for brain targeting [57].

For example, Gabathuler et al. successfully transported the anticancer drug Adriamycin (ADR) to the mouse brain by conjugating it with melanotransferrin or human melanoma antigen p94, which is known to undergo transcytosis through receptor-mediated endocytosis involving low-density lipoprotein receptor-related proteins 1 (LRP1) and 2 (LRP2). One hour after intravenous injection, approximately 0.1% of the administered dose of p94 was reported to have reached the mouse brain (0.25% ID/g, 0.5 g/brain) [58].

In another study conducted by Miyajima et al., transferrin (Tf)-conjugated polyethylene glycol (PEG)-modified (PEGylated) nido-carborane liposomes, internalized via RMT, were synthesized. The study reported an increase in the survival rate of mice to 31 days after treatment, compared to untreated mice with a survival rate of 21 days [59]. Furthermore, Zhang et al. investigated the use of expression plasmids encoding the bioluminescent oxidase enzyme luciferase encapsulated within artificial viruses consisting of 85 nm PEGylated immunoliposomes. *In vivo* testing was performed on a rhesus monkey’s brain, with the monoclonal antibody (mAb) attached to the human insulin receptor (HIR). Following intravenous injection into the monkey brain, the HIRMAb facilitated the transcytosis of the liposome containing the foreign gene across the BBB and the endocytosis across the neuronal plasma membrane. As a result of RMT, gene expression in the monkey brain targeted with the HIRMAb-PIL was approximately 50-fold higher than in the rat brain targeted with the TfRMAb-PIL [60].

AMT, also referred to as the pinocytosis pathway, is initiated by an electrostatic interaction between a positively charged molecule, often a charged segment of a cationic peptide or protein, and the negatively charged surface of the plasma membrane, such as heparin sulfate proteoglycans [61]. In comparison to RMT, adsorptive-mediated transport has a lower affinity but a higher capacity. AMT has garnered significant attention in the development of various innovative drug delivery methods. Cationic proteins or basic oligopeptides, such as cell-penetrating peptides, can be utilized as target molecules for brain-targeted drug delivery based on AMT [62]. Table 1 summarizes the various pathways through which nanocarriers can cross the BBB, and the relevant studies are reviewed below.

There are multiple routes of administration for delivering liposomes to the brain, which will be briefly discussed in this review. *In vivo* and human studies commonly explore administration routes, including intracarotid, intranasal, intracranial, intraperitoneal injections, and convection-enhanced delivery. The carotid artery, located in the neck, is the main blood vessel that supplies blood from the heart to the brain. Intracarotid injection involves directly injecting drugs into the carotid artery. Intranasal injection bypasses the BBB by delivering drugs through the nose to reach the brain. Intracranial injections are an effective method for directly administering drugs to specific regions of the brain. Intraperitoneal injections deliver drugs through the peritoneal route. Convection-enhanced delivery (CED) establishes a pressure gradient at the tip of an infusion catheter implanted in the brain to directly administer drugs into the interstitial spaces of the brain [63].

**Table 1:** Pathways to cross the BBB.

Internalization Pathway	Ligand	Remarks	Reference
Receptor-Mediated Transcytosis	Transferrin (Tf)	-PEGylated liposomes covalently bonded to poly-L-arginine peptide were administered to rats *in vivo* -β-galactosidase activity was reported to be two-fold higher than the control	[60]
	Tripeptide glutathione (GSH)	*-In vivo* study conducted of the administration of ribavirin GSH-PEG liposomes at 50 mg/kg ribavirin -Three-fold increase in ribavirin concentration in the brain in mice treated with GSH-PEG liposomes compared to control	[64]
	Low-density lipoprotein receptor (LDLR)	-The study involved *in vivo* intravenous administration of melanotransferrin (P94) -P94 was accumulated in the mouse brain at an amount over 14 times higher than that of the control group (holo-transferrin)	[65]
Receptor-Mediated Endocytosis	Anti-transferrin receptor IgG	-Gold nanoparticles were conjugated with IgG, and its uptake by brain endothelial cells was studied *in vivo* -Higher accumulation of IgG was observed in mice when compared to the control	[54]
Adsorptive Mediated Transcytosis	Cationized human serum albumin (CHSA)	-CBSA proteins were covalently conjugated to PEGylated liposomes -In vitro studies on porcine brain models exhibited higher uptake in brain capillary endothelial cells (BCEC) when compared to control liposomes	[66]
Adsorptive Mediated Endocytosis	Immunoglobulin γ (IgG)	-Fluorophores conjugated with IgG were administered in vitro to human induced pluripotent stem-cell-derived BECs (iBECs) -Fluorescence was noted to be increased in cells treated with IgG-conjugated fluorophores due to endocytosis of the IgG across the BBB	[67]

### 1.3. Drug Delivery Challenges across the BBB

The BBB poses considerable obstacles in administering therapeutic medications to the brain. One of the foremost obstacles encountered pertains to the tight junctions of the BBB, which consist of intricate protein formations responsible for sealing the intercellular gaps among endothelial cells. This sealing mechanism effectively restricts the passage of various drugs as they have a high degree of selectivity, permitting only specific small molecules that are lipophilic or gaseous to undergo passive diffusion [68]. An additional obstacle arises from the presence of efflux transporters, which actively expel drugs that successfully traverse the BBB, returning them into the systemic circulation, leading to a substantial reduction in drug concentration within the brain tissues. Furthermore, the BBB is equipped with metabolic enzymes capable of metabolizing drugs, thereby reducing their concentration and efficacy [69]. The dimensions and electrostatic properties of pharmaceutical compounds influence their capacity to traverse the BBB. The permeability of large, polar molecules is impeded, whereas small, lipophilic molecules are more likely to pass through. Nevertheless, even these tiny molecules encounter obstacles as a result of the efflux mechanisms. Moreover, the properties of the BBB can undergo alterations due to pathological conditions, which introduce additional complexities in the drug delivery process. Hence, developing strategies for the efficient delivery of drugs across the BBB continues to pose a substantial challenge in the effective treatment of brain tumors [70].

The determinants of central nervous system (CNS) penetration are subject to the influence of various factors that affect the capacity of drugs to get past the BBB to reach the brain tissue. The ability of a drug to traverse the BBB can be influenced by several physiochemical factors, including its dimensions, lipophilic properties, propensity for hydrogen bonding, and molecular weight. The cerebral blood flow rate also impacts the transportation of drugs to the CNS [71]. The metabolism and elimination of pharmaceutical substances within the human body also affect their levels within the circulatory system, thereby impacting their capacity to penetrate the CNS. Certain pharmaceutical substances can interact with proteins present in the circulatory system, thereby diminishing their free concentration and consequently impeding their capacity to traverse the BBB. It is imperative to acknowledge that the BBB exhibits heterogeneity and can vary across different regions of the central nervous system (CNS), causing varying levels of drug penetration into the CNS [72].

**Figure 2:**
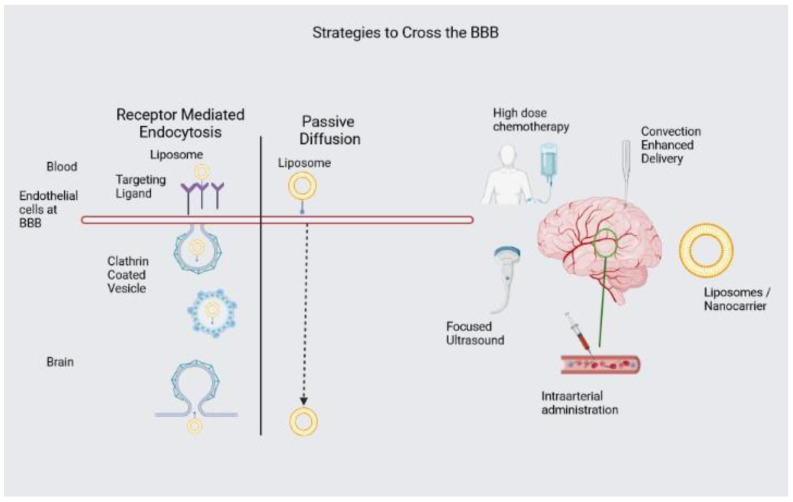
Schematic illustration of strategies to cross the BBB.

Many methodologies are being researched to circumvent the BBB to facilitate drug delivery (Figure 2). High-dose systemic therapy entails the administration of high doses of chemotherapy drugs to augment their concentration within the CNS. This technique presents potential benefits, including a more consistent dispersion throughout the neuraxis. This dispersion is not influenced by the rate or direction of cerebrospinal fluid (CSF) flow, distinguishing it from intrathecal administration or local delivery methods. Nevertheless, this approach has certain inherent limitations, including higher toxicity and the requirement to surpass a certain threshold for BBB permeation to observe a therapeutic effect. BBB disruption enhances the penetration of drugs into the CNS [73]. Infusing a hyperosmotic solution, such as mannitol, is the most clinically utilized approach. This method has been investigated in adult patients with supratentorial malignant gliomas. Nevertheless, this method lacks specificity toward tumors, and the precise levels of drug exposure and concentration remain uncertain. Other techniques that can be employed include the utilization of cytokines, as well as vasoactive substances such as bradykinin, to disrupt the BBB. Direct inhibition of efflux transporters is another strategy to circumvent the BBB. P-glycoprotein (Pgp) functions as a drug efflux pump, thereby restricting the passage of specific drugs across the BBB into the CNS. Its inhibition can be carried out by administering pharmaceutical agents such as cyclosporine A. Nevertheless, the inhibition of Pgp is not limited to the BBB, and can also impact the elimination of drugs from the bloodstream in various other organs. Convection-enhanced delivery (CED) enables the direct administration of pharmaceutical agents into the localized tumor site or specific regions of the brain. As mentioned above, CED uses a pressure gradient that increases drug dispersion within the brain tissue, thereby enabling higher drug concentrations at the intended location. This method provides the benefit of accurate drug administration and the ability to regulate the spatial dispersion of drugs within the brain, in contrast to drugs administered through systemic means. Nevertheless, it is considered invasive and necessitates cautious control to prevent any potential harm to the tissue and the backflow of the infused drug. Focused ultrasound, in conjunction with microbubbles is a non-invasive technique that induces a temporary disruption of the BBB. Lipid-encased microbubbles filled with gas are introduced into the circulatory system. Focused ultrasound is applied to the targeted area of the brain, and the microbubbles undergo oscillation within the acoustic field, resulting in the generation of mechanical forces exerted on the tight junctions of the endothelial cells that form the lining of the vessel wall. The mechanical disruption of the BBB facilitates the enhanced permeability of drugs into the central nervous system. The method employed is reversible and lasts for 4–6 h. The technique enables precise drug administration and reduces harm to unaffected brain tissue. However, utilizing microbubbles has certain risks, and identifying optimal parameters is still under investigation. Intra-arterial (IA) administration is a method of delivering medications directly to the tumor via circulation. Drugs are injected into the arterial vessel responsible for supplying blood to the tumor, thereby facilitating the initial transfer of the drug. However, the application of IA has certain drawbacks, such as the possibility of focal neurotoxicity, the potential for embolism and hemorrhage, and the limited capacity to deliver drugs to a specific area [74].

### 1.4. Drug Delivery Platforms to cross the BBB

Many drugs and drug delivery platforms have shown great potential to cross the BBB. Nitrosoureas drugs have been used to treat cancerous brain tumors for a long time. Carmustine and lomustine are widely utilized nitrosourea compounds in various applications. They have lipid solubility, enabling them to traverse the blood–brain barrier (BBB). Drugs currently available in the commercial market to treat brain tumors include thiotepa, temozolomide, methotrexate, topotecan, irinotecan, cisplatin, and carboplatin. The conjugation of drugs or nanocarriers with ligands that have an affinity towards specific receptors presents a receptor-mediated approach to circumvent the blood–brain barrier (BBB), facilitating their penetration into brain tumors. The expression of the d-glucose transport protein (GLUT) is observed to be significantly higher, approximately 100-fold, in brain tumors compared to transferrin receptors. Glucose molecules conjugated with RGD peptides have also successfully permeated the blood–brain barrier (BBB) [75]. Virus-like particles (VLPs) serve as an immunogenic framework for advancing efficacious therapeutic anti-cancer vaccines targeting brain tumors. Viruses can be imitated in terms of structure and functionality while not inducing any pathogenic effects. These particles can be manipulated to selectively bind to particular cells and tissues, such as brain endothelial cells, and effectively transport therapeutic substances across the blood–brain barrier. Cell-based vehicles, including mesenchymal cells, erythrocytes, neutrophils, and macrophages, have been modified to facilitate the transportation of drugs across the blood–brain barrier (BBB) through a mechanism known as cell-mediated transport (CMT). This process entails actively transporting diverse bioactive compounds across the cellular membrane. The aforementioned pathway relies on energy and typically involves transporting small hydrophilic molecules. Nanoparticle-based drug delivery systems, discussed in the next section, have also been used to successfully deliver drugs across the BBB [76]. As the scope of this review primarily focuses on using liposomes to treat brain cancers, we would like to direct the readers to the following references for more detailed information [73,74,75,76]. 

## 2. Nanoparticle-Based Drug Delivery Systems

Nanoparticle drug delivery systems (DDSs) have garnered significant interest in the search for new, more effective, and less invasive approaches to cancer treatment. This is primarily due to their capacity to reduce the side effects commonly associated with conventional chemotherapy, such as lack of specificity and premature drug release [77]. They involve the utilization of nanocarriers comprised of non-toxic monomers and polymers that exhibit high physical and chemical stability, as well as biocompatibility. These nanocarriers can be modified to specifically target receptors on tumor cells, enabling precise and site-specific drug delivery. Additionally, nanoparticle DDSs can be designed to respond to various stimuli, including temperature, heat, light, pH, and ultrasound. Most nanoparticle DDSs exhibit biocompatibility, biodegradability, and low toxicity, although exceptions exist, such as certain metal/metal oxide nanoparticles whose biodegradability depends on the coating and synthesis methods employed [77,78]. Several nanoparticle DDSs have been extensively studied, including liposomes, polymeric nanoparticles, exosomes, metal–organic frameworks (MOFs), quantum dots (QDs), dendrimers, and hydrogels. These nanocarriers represent diverse structures and properties that offer unique advantages for drug delivery applications. It is important to note that this list is not exhaustive, as ongoing research continues to explore and develop new nanoparticle-based drug delivery systems. 

A common class of nanocarriers includes micelles, which are composed of amphiphilic molecules, including block copolymers, which undergo self-assembly to form acore–shell structure in an aqueous solution. The core of the micelles is composed of the hydrophobic blocks of the copolymers, while the shell is formed by the hydrophilic blocks. Hydrophobic drugs can be incorporated into the core, while hydrophilic drugs can be loaded into the shell. The loading of drugs into micelles can occur during their formation or at a later stage using methods such as dialysis or film hydration. Like liposomes, micelles can respond to both intrinsic and extrinsic stimuli [79]. Similarly, hydrogels are composed of a significant water content embedded within a network of cross-linked polymers. These networks are typically formed through crosslinking during or after radical chain polymerization. Hydrophilic drugs can be absorbed into the hydrogels through diffusion via openings in the polymer network or by incorporating the therapeutics during the polymerization process. The responsive behavior of hydrogels, such as expansion and contraction, can be tailored to pH and temperature stimuli, enabling the retention or expulsion of drugs. Table 2 provides an overview with references to other common nanocarriers utilized in smart drug delivery systems (DDSs).

### 2.1. Liposomes

Liposomes are spherical vesicles with an aqueous interior surrounded by one or more phospholipid bilayers, with sizes ranging from less than 0.5 μm to over 100 μm. They are mainly composed of phospholipids, which can be classified into glycerophospholipids and sphingomyelins. Glycerophospholipids have a hydrophilic glycerol head group that is esterified to hydrophobic alkyl side chains [86]. This group includes phosphatidylcholine (PC), phosphatidylethanolamine (PE), phosphatidylserine (PS), phosphatidylinositol (PI), phosphatidic acid (PA), phosphatidylglycerol (PG), and cardiolipin. Glycerophospholipids, such as dimyristoyl, dipalmitoyl, or stearoyl PC, are derived from the variation in the length of the nonpolar alkyl moieties. Additionally, the type of bonding (ether or ester) between glycerol and the aliphatic chains results in distinct glycerophospholipids [87]. These bilayers of phospholipids are composed of hydrophilic heads and hydrophobic tails. The number of bilayers present in a liposome defines its lamellarity. The bilayer structure allows for the entrapment of hydrophobic drugs within the bilayer itself, while hydrophilic drugs can be encapsulated in the aqueous interior. Liposomes can be designed to have sizes suitable for accumulation at tumor sites. Additionally, their surfaces can be easily modified by conjugating targeting moieties, enabling site-specific drug delivery [88].

Drugs can be incorporated into liposomes using either passive or active loading methods. In the passive loading of water-soluble drugs, the drug is dissolved in the aqueous solution to form the liposome. For passive loading of hydrophobic drugs, the drug is dissolved with the lipids in an organic solvent. The solvent is then evaporated, leaving behind lipid bilayers containing the drug. In active loading, the liposomal vesicle is initially formed without the drug. Then the drug is introduced into the liposome through mechanisms such as the pH-gradient technique or other transmembrane gradients, allowing it to diffuse into the liposome. They are known for their inherent stability and slow release of drugs. To enhance drug release, various stimuli such as ultrasound, pH, temperature, light, and redox have been developed [89].

Covalently conjugating polyethylene glycol (PEG) chains are widely recognized as an effective method to enhance liposome stability and prolong their circulation half-life *in vivo*. PEGylation, for instance, has been demonstrated to inhibit the clearance of liposomes by the mononuclear phagocytic cells in the liver and spleen by preventing the adsorption of circulating protein opsonins onto liposome surfaces. As a result, PEGylation extends the duration during which liposomes and their encapsulated therapeutics remain in the bloodstream. However, PEGylation gives rise to a predicament known as the “PEG dilemma.” The presence of PEG chains can impede the efficient uptake of liposomes by cancer cells through endocytosis and transcytosis. The length of the PEG chains can hinder the binding of targeting ligands on liposomes to complementary cell surface receptors. Moreover, the PEG chains may hinder the effective release of the drug at the tumor site and impede endocytosis by the plasma membrane of tumor cells, thereby reducing the overall efficiency of the liposomes [90]. 

PEGylation can also lead to a phenomenon called accelerated blood clearance (ABC), wherein repeated administration of PEGylated liposomes results in increasingly rapid clearance from the systemic circulation due to macrophage uptake. In the field of drug delivery, both PEGylated and non-PEGylated liposomes have received clinical approvals. An exemplary PEGylated liposomal formulation is Doxil^®^, which encapsulates the anti-cancer drug doxorubicin. Doxil^®^ is licensed for the treatment of Kaposi’s sarcoma, ovarian cancer, breast cancer, and multiple myeloma. The addition of cholesterol to liposome formulations is essential for their stability. Cholesterol impacts various liposome properties, including fluidity, permeability, membrane strength, elasticity, stiffness, transition temperature, and drug retention [91,92].

Notably, there are certain drawbacks associated with using liposomes as chemotherapeutic nanocarriers. One concern is their potential for physical and chemical instability, primarily attributed to the propensity of liposomes to accumulate. The ester link in the acyl group of the phosphatidylcholine molecule within the bilayer may undergo hydrolysis, resulting in the formation of lysophosphatidylcholine. This can compromise the stability of liposomes and lead to premature drug release. If not PEGylated, liposomes may be rapidly eliminated from the circulatory system through the monocyte–macrophage system [93,94]. While the PEGylation of liposomes proved to reduce clearance by the reticuloendothelial system (RES), it is worth noting that the presence of PEG on liposomes inhibits their fusion with cell membranes, in contrast to non-PEGylated liposomes [95]. Moreover, liposomes pose challenges in sterilization, as the phospholipid bilayer is sensitive to heat. Additionally, the production costs of liposomes can be high, and the industrial-scale functionalization of liposomes can be complex. Furthermore, the long-term storage of liposomes can also be a concern [96,97].

#### 2.1.1. Preparation and Functionalization 

Conventional methods for preparing liposomes can be broadly categorized into hydration methods, detergent removal methods, and solvent injection methods. Among these, hydration methods are the most commonly used. There are three general protocols within hydration methods:

- Direct dissolution: In this method, lipids are directly dissolved in an aqueous medium, forming liposomes.
- Thin film hydration: A thin lipid film, formed by evaporating lipids dissolved in an organic solvent, is deposited on a glass substrate. The film is then hydrated using an aqueous solution, leading to the formation of liposomes.
- Electroformation: This method involves applying an external electric field during the hydration step of the thin lipid film. The electric field enhances water influx between the bilayer sheets of the thin film, resulting in the formation of giant unilamellar vesicles (GUVs).

It is necessary to apply external mechanical forces such as sonication or extrusion to break up multilamellar dispersions into unilamellar populations if unilamellar liposomes are desired [98].

The thin-film hydration method, also known as the Bangham method, is one of the oldest and most widely used techniques for liposome preparation [99,100]. Drawbacks of this conventional preparation method include low entrapment efficiencies (most of the drug solution never becomes encapsulated), difficulty in upscaling production, and the heterogenous size distribution of the liposomal dispersion [101]. Therefore, recent hydration methods are being developed to counteract these limitations. For instance, packed-bed hydration was introduced by Sundar and Tirumkudulu [102], where the drying, followed by subsequent hydration of the lipid–organic solvent mixture, is carried out in a packed bed filled with asymmetric roughened colloidal alumina particles. The produced liposomes showed increased encapsulation efficiencies. While the size distribution of the produced liposomes was independent of the packing density and size, it did depend on the packing material’s porous structure. Another method was proposed by Skalko-Basnet et al. [103], where the lipid–organic mixture was prepared by dissolving dry lipid powders with mannitol in chloroform; then, the mixture was spray-dried. The thin film formed from spray-drying was amorphous; thus, vesiculation occurred simultaneously upon agitated hydration and produced liposomes ranging in size from 300 to 500 nm. The size of the liposomes depended on the volume of the aqueous mixture used in the hydration step. Similarly, Li and Deng [104] tested freeze-drying the lipids and then hydrating the lipid film with an aqueous medium, which resulted in a homogenously distributed liposomal solution. The size of the produced liposomes depended on the ratio of the lipids to the organic solvent used in preparing the initial mixture. 

Aside from the previously discussed techniques, synthesis in microfluidics has emerged as a promising technique that allows reproducibility, usability, stability, and enhanced process control [105]. In their pioneering work, Lin et al. [106] introduced the use of a microfluidic device to prepare liposomes. Their device featured a channel where an aqueous buffer was injected over a thin lipid film. However, this early microfluidic approach had some drawbacks. The resulting liposomes were not unilamellar and exhibited a lack of size homogeneity. Additionally, the encapsulation efficiency of the liposomes was considerably low, and the processing time was longer than traditional hydration methods. Despite these limitations, this work marked an important milestone in using microfluidic devices for liposome fabrication. Their efforts laid the foundation for subsequent researchers to explore and refine the use of microfluidic techniques in the production of liposomes.

Subsequently, several innovative microfluidic techniques have been developed for liposome fabrication. Ota et al. [107] introduced a method based on transient membrane ejection, where a lipid bilayer is formed and then disrupted by a continuous fluid stream, resulting in smaller vesicles. The size of the produced liposomes can be effectively controlled by controlling the flow rate of the aqueous buffer. In another approach by Jahn et al. [108], three streams were mixed in a microchannel. The central stream contained phospholipids dissolved in alcohol, while the side streams contained aqueous solutions. As the alcohol diffused into the aqueous phase, the lipids self-assembled into liposomes ranging in size from 50 to 150 nm. Pautot et al. developed a droplet emulsion transfer technique in a microfluidic chip to form unilamellar liposomes. Stabilized by phospholipids, a water-in-oil emulsion was created, and the droplets were transferred to the aqueous phase. As the droplets traversed the interface between the organic and aqueous phases, they accumulated an additional lipid layer, forming liposomal structures. Additionally, Kastner et al. devised a technique relying on the chaotic advection of a staggered herringbone micromixer (SHM). In this method, separate streams of lipid solution and aqueous buffer were injected into the device at controlled flow ratios. The fluid streams mixed, stretched, and folded over the channel surface due to chaotic advection, promoting mass transfer and producing liposomes of controllable size and polydispersity [109]. These diverse microfluidic techniques offer precise control over liposome size and enable the production of liposomes with enhanced homogeneity, addressing some of the limitations of conventional methods.

#### 2.1.2. Functionalization Approaches 

Targeting ligands, such as proteins, antibodies, carbohydrates, aptamers, and polypeptide sequences, can be conjugated to the surface of liposomes through covalent or non-covalent bonds. To anchor the ligands onto the liposomal surface, various molecules with specialized functional groups are employed. Covalent bonding of ligands to liposomes can be achieved using different coupling techniques, including thioether bonds, hydrazone bonds, carboxamide bonds, amide bonds, and disulfide bonds [110]. Among these techniques, thioether bonds are widely utilized for functionalizing liposomes due to their rapid reaction rate at neutral pH and the formation of relatively stable bonds. Phospholipids containing maleimide groups can react with ligands possessing thiol groups, such as Fab fragments, forming stable covalent bonds. To convert the ligand’s amines into free thiols (sulfhydryl groups), Traut’s reagent is used, which can then react with the maleimide groups on the substrate surface [111]. Carboxamide bonds offer another option for conjugating ligands to liposomal surfaces. This method involves using an anchor molecule with carboxylic acid end groups. In the presence of carbodiimides, the coupling reaction generates an acyl amino ester, which subsequently reacts with the primary amine of the ligand to form an amide bond [112]. 

Another coupling technique employed is the disulfide bond, which involves the reaction between thiol groups on the surface substrate and those on the ligands. Disulfide bonds, also known as SS-bonds or disulfide bridges, are commonly formed by coupling two thiol groups (also referred to as sulfhydryl groups) under oxidizing conditions. Thiol-disulfide exchange is another chemical reaction that occurs when a thiolate group (-S-) attacks a sulfur atom of a disulfide bond (-S-S-). This process disrupts the initial disulfide bond and releases the second sulfur atom as a new thiolate, eliminating the negative charge. Disulfide bond formation is a frequently used conjugation linker; however, it is prone to homocoupling, where two identical thiols can form the same disulfide bond, leading to reduced selectivity. Additionally, disulfide bonds can be cleaved by reducing agents, which can be advantageous for specific applications [113]. 

A hydrazone bond can be utilized to attach antibodies to carbohydrate ligands by introducing hydrazide groups onto the liposomal surface. The carbohydrate moieties can be oxidized either by galactose oxidase or sodium periodate. After the oxidation process, the resulting oxidized antibodies can be coupled directly to a lipid bilayer containing a hydrazide-hydrophobic anchor, such as lauric acid hydrazide, or to the hydrazide groups located at the distal end of the PEG chains of sterically stabilized liposomes [114]. 

Non-covalent physical interactions can also serve as a method to immobilize biomolecules on the surface of nanoparticles. These interactions rely on attractive forces such as electrostatic interactions, hydrogen bonding, and van der Waals forces to form complexes [115]. While this approach has demonstrated effectiveness in various applications, it faces challenges in controlling the orientation of the ligand on the surface. The orientation of the ligand can impact its stability and activity. For instance, if a tertiary protein adheres to the surface through hydrophobic interactions, it may undergo slight structural changes that could affect its activity. Additionally, these interactions are highly sensitive to the physiochemical conditions of the hosting buffer. Changes in ionic strength, pH, or the isoelectric point of the ligand can destabilize electrostatic interactions, potentially leading to the detachment of the ligand from the nanoparticle’s surface [116].

After accumulating in the tumor’s leaky vasculature, either through the enhanced permeability and retention (EPR) effect or active biological targeting, liposomes need to be able to release their cargo in a controlled, timely, and efficient manner. Stimuli-responsive liposomes have garnered significant attention in recent years for their therapeutic applications. These liposomes are designed to maintain stability while circulating in the body but can release their contents upon exposure to specific stimuli. The triggering mechanism can either originate internally within the targeted tissue, such as changes in pH, enzymatic levels, or redox levels, or be external stimuli like temperature, ultrasound, light, magnetic fields, or electric fields [117].

Yuba et al. conducted a study where they designed a pH-sensitive liposomal system loaded with the anti-cancer drug bleomycin [118]. The results demonstrated that these liposomes exhibited sensitivity to low pH levels at tumor sites and a 2.5-fold increase in drug release in response to pH changes. In another study by Lajunen et al., a light-triggered liposomal system was developed and loaded with the light-sensitive dye indocyanine green. The study revealed that the structure of the liposomes was significantly influenced by the localization of the light-triggering agent, which could be advantageous for drug delivery purposes [119]. Additionally, Zeng et al. synthesized thermosensitive liposomes containing the chemotherapeutic agent oxaliplatin. The findings indicated that at a temperature of 42 °C, over 90% of the oxaliplatin was released within 10 min of administration. However, at temperatures below 39 °C within 60 min, less than 15% of the drug was released. Furthermore, the anti-tumor activities of the oxaliplatin-loaded liposomes at a dose of 2.5 mg/kg were comparable to those of non-thermosensitive liposomes and oxaliplatin injection at a dose of 5 mg/kg [120]. Despite the increasing use of triggering modalities for initiating release from liposomal nanocarriers, the translation of these approaches into real-life brain-targeted applications is impeded by the inherent complexity of the brain structure.

#### 2.1.3. Advantages of Liposomes over Other Nanocarriers

Liposomes possess numerous characteristics that render them versatile and adaptable nanocarriers for drug delivery. The unique structure of liposomes enables them to encapsulate both hydrophilic and hydrophobic drugs, thereby augmenting their adaptability. They can enhance the stability and bioavailability of chemotherapeutic agents, while mitigating their toxicity by impeding direct interaction with healthy cells. Moreover, liposomes can be readily customized to facilitate targeted drug delivery, thereby enhancing their efficacy. Additionally, their safety profile has been well-established, as evidenced by the approval of numerous liposomal drugs for clinical utilization. As a result, liposomal formulations find applications in various targeted drug delivery scenarios, encompassing respiratory and ocular diseases, cancer chemotherapy, cancer vaccines, pulmonary disorders, fungal and viral infections, gene therapy, and even cosmetics [121]. Liposomes have been successfully utilized to deliver drugs, protein molecules, nucleic acids, and imaging agents. They are considered suitable nanocarriers for targeted drug administration due to their biocompatibility, amphiphilic nature, and non-ionic properties. Liposomes facilitate sustained drug release, prevent medication oxidation and premature degradation, and enhance drug efficacy. Liposomes can also incorporate passive and active targeting strategies, which are particularly crucial in cancer therapy. Through passive targeting, liposomes can accumulate in tumor cells while minimizing accumulation in healthy tissues, such as the kidneys and heart (known as the side avoidance effect). This approach enhances drug stability, reduces systemic toxicity, and improves local therapeutic effectiveness [122].

Liposomes offer the advantage of active targeting by incorporating site-specific or tissue-specific ligands. This allows them to accumulate in specific tissues and subsequently release the drug or payload exclusively within the targeted area. As a result, liposomes minimize the potentially harmful effects of the drug on non-targeted tissues. Additionally, liposomes, owing to their structural similarity to cell membranes, can easily permeate most biological membranes. This property enhances the efficacy of the drug and amplifies its therapeutic benefits [123].

*In vitro* experiments conducted on RG-2 tumor cells in rats revealed that the average size of fenestrations and inter-endothelial gaps were measured to be 48 nm and 1 μm, respectively, making the BBB susceptible to liposomes, and the EPR effect facilitates their selective accumulation in tissues affected by brain tumors. The liposomes successfully extravasated through the BBB to the tumor, substantially reducing tumor size [124]. A study by Krishna and colleagues involved synthesizing immunoliposomes conjugated with the monoclonal antibody transferrin (OX26 mAb) and encapsulating dioxin. The study reported an increase in dioxin uptake by a factor of 25 in rat endothelial cells due to the inhibition of the P-gp efflux transporters, allowing more of the drug to accumulate in tumor tissue [125]. Another study by Lee et al. reported that liposomes encapsulating P-gp efflux inhibitor PSC 8339 and doxorubicin were able to successfully penetrate the BBB and accumulate into tumors via RME as well as the action of the efflux inhibitors. Therefore, liposomes can be advantageous in circumventing the challenges posed by the BBB. Liposomes have a high drug-loading capacity, unlike micelles and other nanocarriers, which have a lower drug capacity [126].

Cationic or anionic liposomes can be synthesized based on the specific requirements of the drug to be delivered and the characteristics of the blood–brain barrier (BBB) in the targeted condition. Cationic liposomes have surfaces that carry a positive charge, enabling them to interact with the negatively charged endothelial cells of the BBB. This interaction can potentially enhance the internalization of cationic liposomes into the brain through adsorptive-mediated transcytosis. They also can interact with negatively charged constituents of the BBB, which may augment their capacity to traverse the barrier. Conversely, anionic liposomes have surfaces that have a negative charge [127]. Although they do not exhibit the same level of direct interaction with the BBB as cationic liposomes, they can still demonstrate efficacy in facilitating drug delivery to the brain. They can utilize RMT mechanisms, which aid in the transportation of the liposomes across the barrier. Moreover, they are less toxic, in general, to the human body, and the rate of release from the liposomes can be effectively controlled, giving them a clear advantage over other nanocarriers for drug delivery to the brain [128].

## 3. Liposomal Treatments for Brain Tumors

### 3.1. Preclinical Studies

**Figure 3:**
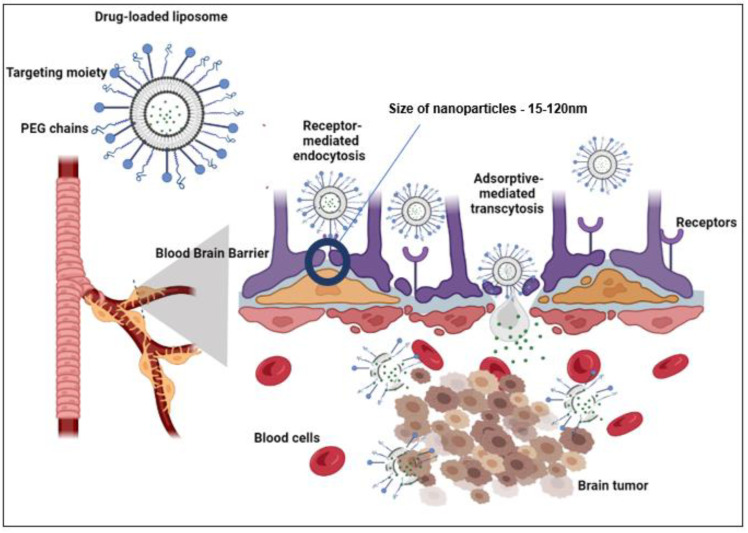
Schematic illustration of liposomal drug delivery across the BBB.

Liposomes have the potential to enhance drug delivery to brain tumors and improve treatment effectiveness. They exert their therapeutic effect by releasing their cargo in specific regions of the tumor’s vasculature and extravascular space (Figure 3). Through targeted delivery, liposomes can be directed to specific areas of the BBB or glioblastoma multiforme (GBM) tumors to deliver anti-cancer drugs. This section discusses several studies primarily aimed at translating these promising formulations into preclinical settings.

In a study conducted by Gaillard et al. [129], the penetration of a commercially available glutathione DOX–PEGylated (2B3–101) liposomal formulation through the BBB was investigated *in vitro* and *in vivo* using mice. The research demonstrated improved tumor regression in a murine model due to the increased delivery of doxorubicin from the targeted liposomes. In comparison to free DOX, the pharmacokinetic profile showed that both 2B3–101 and PEGylated liposomal DOX were taken up at a higher rate *in vitro,* following incubation with 450 µg HSPC per mL of both liposomal formulations for 1 to 5.5 h. Immediately after administration, PEG-lipo-DOX and 2B3-101 exhibited similar concentrations in blood plasma; however, there was a significant difference in plasma concentrations 21 h after treatment (*p* = 0.0187). Biodistribution data revealed that DOX concentrations were highest in the brain following treatment with 2B3–101 (*p* = 0.0039), approximately three times higher than in animals given free doxorubicin (*p* = 0.01) and 1.5 times higher than in animals given PEGylated liposomal DOX. The median survival time for animals receiving saline treatment was 13 days. In contrast, animals receiving PEG-lipo-DOX had a median survival time of 15.5 days (*p* = 0.05 compared to saline). Animals receiving 2B3–101 had a median survival time of 18 days (*p* = 0.05 and *p* < 0.001 compared to PEGylated liposomal DOX and saline, respectively). These results highlight the potential usefulness of targeted liposomal formulations, such as 2B3–101, in improving drug delivery to brain tumors and enhancing treatment efficacy. However, it is important to note that this study was conducted in preclinical models, and further research, including clinical trials, would be necessary to determine the safety and efficacy of these liposomal formulations in human patients. Nonetheless, these results demonstrate the potential usefulness of targeted liposomal formulations, such as 2B3–101, in improving drug delivery to brain tumors and enhancing treatment efficacy. They provide a promising foundation for future investigations and clinical trials aimed at developing effective liposomal-based therapies for brain cancer patients.

Aryal et al. [130] conducted a study to investigate the effects of focused ultrasound (FUS)-induced disruption of the BBB after the administration of liposomal doxorubicin (Lipo-DOX) *in vivo*. The study involved nine rats, five receiving Lipo-DOX combined with FUS, while the remaining four received FUS only. Subsequent examination of the brain tissue revealed a significant increase in the concentration of doxorubicin in the Lipo-DOX group (4.8 ± 0.5 µg/g) compared to the FUS-only group (0.819 ± 0.482 µg/g). These findings indicate that combining Lipo-DOX administration with FUS-induced BBB disruption leads to enhanced drug delivery to the brain tissue. The temporary disruption of the BBB caused by FUS allows the increased penetration of liposomal doxorubicin into the brain, resulting in higher drug concentrations at the target site.

In another study, Muthu et al. [131] developed liposomes coated with d-α-tocopheryl polyethylene glycol 1000 succinate (TPGS), a PEGylated vitamin E, and loaded them with docetaxel (DTX) as a model drug for the improved treatment of brain tumors. The liposomes were synthesized using the solvent injection method, and their mean particle size ranged from 126 to 191 nm. The drug encapsulation efficiency was 64.1 ± 0.6% after 24 h of dialysis in PBS. After 24 h of cultivation with C6 glioma cells, the IC50 (drug concentration required to kill 50% of cells) was determined to be 7.04 ± 1.05, 31.04 ± 0.75, 7.70 ± 0.22, and 5.93 ± 0.57 μg/mL for Taxotere^®^, naked liposomes, PEG-coated liposomes, and TPGS-coated liposomes, respectively. The study concluded that TPGS-coated liposomes exhibited significant improvements *in vitro* compared to PEG-coated liposomes. These studies contribute to understanding liposomal drug delivery systems in the context of brain tumor treatment. Aryal et al. demonstrated the potential of combining liposomal DOX with FUS-induced BBB disruption to enhance drug concentration in the brain, while Muthu et al.’s research highlighted the advantages of TPGS-coated liposomes in terms of drug encapsulation efficiency and improved efficacy *in vitro*. However, further studies, including clinical trials, would be necessary to assess the safety and efficacy of these approaches in human patients.

In a similar investigation, theranostic liposomes decorated with RGD-TPGS and co-loaded with docetaxel and QDs were tested *in vivo* on Charles Foster rats [132]. These liposomes had a mean particle size of 175.6 ± 3.2 nm. The release of the drug from the liposomes after 72 h of dialysis in PBS at pH 7.4 was 68.41 ± 3.56%. After 2 h and 4 h of therapy, the RGD-TPGS-decorated theranostic liposomes exhibited 6.47-fold and 6.98-fold higher efficacy than Docel^®^ *in vivo*. The liposomes were internalized into tumor cells through RME, where the liposomes were synthesized by solvent injection method. Moreover, the RGD-TPGS-decorated theranostic liposomes effectively reduced ROS production and showed no evidence of brain injury or edema in brain histopathology. Targeted liposomes, such as those decorated with RGD-TPGS, show promise in enhancing the efficacy and specificity of drug delivery to brain tumor cells. Future research should focus on developing even more precise targeting strategies by exploring other ligands or receptor-specific molecules that can further improve the selectivity of liposomes toward tumor cells.

In another study conducted by Lu et al., thermosensitive magnetic liposomes (TML) encapsulating Camptosar (CPT-11), coated with magnetic Fe_3_O_4_ nanoparticles, and conjugated with Cetuximab (CET) were synthesized. These liposomes demonstrated high biocompatibility and enhanced intracellular uptake by human primary glioblastoma cells (U87) when exposed to a high-frequency alternating magnetic field (AMF). *In vivo* studies demonstrated the efficacy of these liposomes, where the tumor sizes reported for the experimental group on days 14, 17, and 21 after liposome administration were 1.9 mm^3^, 27.1 mm^3^, and 76.3 mm^3^, respectively, compared to the control group with tumor sizes of 29.1 mm^3^, 105.9 mm^3^, and 109.5 mm^3^, respectively. The liposomes were internalized into the cells using the RME pathway and were synthesized using the freeze–thaw method [133]. These studies highlight the potential of using targeted and theranostic liposomes for drug delivery in brain tumor treatment. The RGD-TPGS-decorated liposomes demonstrated enhanced efficacy and specificity, with the ability to reduce ROS production and minimize brain injury. Similarly, the thermosensitive magnetic liposomes exhibited improved intracellular uptake and significant tumor size reduction *in vivo*. These findings contribute to the development of novel strategies for targeted drug delivery and theranostics in brain tumor therapy. Likewise, the co-loading of drugs within liposomes, as demonstrated in the studies, presents opportunities for combination therapies. Combinations of chemotherapeutic agents, targeted therapies, immunotherapies, or other treatment modalities can be encapsulated within liposomes to create synergistic effects and enhance therapeutic outcomes. While the studies mentioned provide promising results in preclinical models, further research is necessary to validate the safety, efficacy, and scalability of these liposomal formulations for clinical use. Future perspectives may involve conducting clinical trials to evaluate the performance and therapeutic benefits of targeted and theranostic liposomes in patients with brain tumors, aiming to improve treatment outcomes and patient quality of life. Moreover, further research may focus on identifying optimal drug combinations and exploring the potential synergies that can be achieved with theranostic liposomes.

Another group developed TMZ-loaded liposomes and utilized ultrasound-mediated BBB permeabilizing technology to achieve localized delivery of these nanoparticles into GBM [134]. In an *in vitro* BBB model, TMZ-liposomes demonstrated significantly higher efficacy in killing C6 tumor cells when combined with ultrasound (US) irradiation compared to free TMZ alone. In mice treated with US-mediated BBB opening, TMZ-liposomes were transcytosed more efficiently due to FUS-induced BBB disruption. Focused ultrasound penetrates the skull and generates microbubbles in the blood, leading to various cavitation effects near the BBB and within the GBM. This resulted in substantial suppression of tumor growth and prolonged animal survival. The liposomes were internalized into the cells using the RMT pathway and were synthesized using the thin-film hydration method [135]. The localized delivery achieved with the application of TMZ-loaded liposomes with US irradiation showed superior tumor growth suppression and increased animal survival. The combination of TMZ-loaded liposomes with ultrasound-mediated BBB permeabilization demonstrated enhanced localized drug delivery to GBM tumors. This approach uses focused ultrasound to temporarily disrupt the BBB, allowing liposomes to cross into the brain. Future research may focus on optimizing the ultrasound parameters and developing strategies to improve the efficiency and safety of this technique for clinical applications.

In a study by Gao et al. [136], DOX-containing liposomes conjugated with both transferrin (Tf) and folate were developed to enhance the transport of DOX across the BBB and into brain gliomas. The liposomes had an average particle size of 180 nm ± 12.5 nm. DOX was efficiently entrapped in the Tf-DOX-liposomes with an encapsulation efficiency of 94.5% ± 0.5%. The liposomes were able to cross the BBB through receptor-mediated endocytosis. The dual-targeting strategy significantly improved the survival of mice with brain tumors and reduced cytotoxicity compared to the groups treated with the free drug. Similarly, the dual-targeting approach employing DOX-containing liposomes conjugated with transferrin (Tf) and folate has shown enhanced transport across the BBB and improved therapeutic outcomes in models of brain glioma. This dual-targeting strategy exploits the overexpression of Tf and folate receptors on tumor cells to enhance liposome uptake. Further investigations may explore the potential of other targeting ligands or receptor-specific molecules that can improve the efficiency of liposomal transport across the BBB and increase tumor accumulation. The studies mentioned provide promising results in preclinical models, highlighting the potential of liposomal drug delivery for brain tumor therapy. Future perspectives may involve conducting rigorous preclinical studies to further evaluate the safety and efficacy of liposomal formulations. Subsequently, clinical trials should be conducted to assess the performance of liposomes in patients with brain tumors and to determine their impact on patient outcomes.

A study conducted by Ghaferi et al. investigated the anti-cancer efficacy of PEGylated liposome nanoparticles (PEG-Lip) loaded with the chemotherapy drugs doxorubicin and carboplatin (CB) for treating brain cancer. The synthesized liposomes had a size of 212 nm ± 10 nm. *In vitro* release studies at pH 7.4 demonstrated controlled release of the encapsulated drugs, with PEG-Lip-DOX, PEG-Lip-CB, and PEG-Lip-DOX/CB releasing 79.3%, 76.7%, and 69.3% of the loaded drugs, respectively, in 52 h. PEG-Lip-DOX/CB exhibited 1.5-fold higher cytotoxicity and 1.3-fold higher reactive oxygen species (ROS) generation compared to Lip-DOX/CB. In glioblastoma-bearing rats, PEG-Lip-DOX/CB significantly increased survival time by 23.1% and 10.2% compared to DOX + CB and Lip-DOX/CB, respectively. The liposomes were internalized into the cells using the RME pathway and were synthesized using the thin-film hydration method [137]. The improved cytotoxicity and increased reactive oxygen species (ROS) generation observed with PEG-Lip-DOX/CB suggest a potential synergistic effect of the drug combination within the liposomal formulation. Moreover, the significant increase in survival time in glioblastoma-bearing rats treated with PEG-Lip-DOX/CB highlights the therapeutic potential of this liposomal system.

In another study, liposomes coupled with various cell-penetrating peptides (CPPs) were investigated as a gene vector delivery system across the BBB. Liposomes conjugated with CPPs and transferrin (Tf) ligands were synthesized, and their impact on liposome transport capacity and transfection effectiveness in brain endothelial cells was studied. The liposomes had a size of 155 nm and an encapsulation efficiency of 84.6%. *In vitro* studies demonstrated increased uptake in brain endothelial cells, with uptake levels reaching 33% and 71% after 1 h and 4 h of incubation, respectively. *In vivo* biodistribution studies in mice showed that 7.7% of the administered dosage was able to penetrate the BBB and accumulate in brain tumors via endocytosis [138]. The increased uptake in brain endothelial cells and the accumulation of liposomes in brain tumors demonstrated in *in vitro* and *in vivo* experiments indicate the potential of these liposomes as gene delivery vectors for brain cancer treatment. These studies emphasize the versatility and potential of liposomal drug delivery systems for brain cancer therapy. The ability to optimize liposome characteristics, incorporate targeting ligands, and achieve controlled release of drugs provides opportunities to overcome the challenges associated with brain tumor treatment. Further research and development in this field hold promise to translate these findings into clinical applications, offering improved treatment options for patients with brain cancer.

Kang et al. [139] reported an innovative method for modifying liposomes that encapsulate docetaxel (DTX). The modification involved using R17217, a transferrin receptor-specific antibody, and muscone, a constituent found in Chinese medicinal products. The liposomes had a radius of 123.5 ± 1.3 nm, an encapsulation efficiency of 87.56 ± 5.05%, and a reported conjugation efficiency of R17217 of 52.68%. The liposomes remained stable even after 24 h of incubation, and they exhibited sustained DTX release, with RI-LP-DTX showing a slower release profile compared to PEG-LP-DTX. In a mouse xenograft GBM model, these modified liposomes demonstrated enhanced penetration through the BBB and longer circulation time, resulting in approximately two-fold higher delivery to brain tumors. Modifying liposomes with specific ligands offers a promising approach for targeted drug delivery in brain tumor therapy. The modified liposomes showed enhanced penetration through the BBB, longer circulation time, and increased drug delivery to brain tumors. These findings suggest that the conjugation of targeting ligands to liposomes can enhance their efficacy in brain tumor treatment by facilitating specific and efficient uptake into tumor cells. In addition, Li et al. [140] developed liposomes containing elemene (ELE) and cabazitaxel (CTX) and modified the liposomes by conjugating them with transferrin (Tf) or embedding RG2 glioma cell membrane proteins. The liposomes had a mean size of 135.1 ± 4.2 nm, and the encapsulation efficiency was 99.8% for ELE/CTX liposomes and 99.1% for Tf-ELE/CTX liposomes. No significant changes in particle size were observed after 7 days of storage at 4 °C. *In vivo* experiments demonstrated that Tf-ELE/CTX liposomes exhibited enhanced anti-tumor effects, leading to increased survival time and reduced tumor volume in mice by approximately 1.5-fold compared to control solutions of CTX and ELE. The modified liposomes exhibited enhanced anti-tumor effects, as evidenced by increased survival time and reduced tumor volume in mice. The successful modification of liposomes with Tf or glioma cell membrane proteins improved their selectivity and specificity, leading to improved therapeutic outcomes. Future research in this field may involve exploring the potential of combining different ligands or incorporating multiple targeting strategies to further enhance the specificity and efficacy of liposomal drug delivery systems. Additionally, investigations into the long-term safety, scalability, and translation of these modified liposomes to clinical applications would be valuable for their potential use in brain tumor therapy.

Another *in vivo* study by Guo et al. [141] demonstrated that DOX-loaded PEGylated liposomes functionalized with tumor necrosis factor-related apoptosis-inducing ligand (TRAIL) exhibited a greater anti-GBM impact compared to free drugs or non-modified liposomal drugs alone. This suggests that incorporating TRAIL onto liposomes may enhance their ability to induce apoptosis in GBM cells, potentially leading to improved therapeutic outcomes. The study highlights the importance of targeting specific pathways involved in tumor cell death to enhance the efficacy of liposomal drug delivery systems in brain cancer treatment. In a separate investigation [142], oxaliplatin-loaded liposomes (Lipoxal) were evaluated *in vivo* using an intracranial F98 glioma model, and their performance was compared to free DOX. The results showed that the concentration of oxaliplatin at the glioma site was 2.4 times higher for Lipoxal compared to free oxaliplatin. Additionally, the median survival time of the rats increased from 21.0 ± 2.6 days to 29.6 ± 1.3 days, indicating that the liposomal formulation significantly reduced the toxicity of free oxaliplatin [143]. This demonstrates that liposomes can enhance the delivery and accumulation of the drug within the tumor, potentially leading to increased therapeutic efficacy. Moreover, the liposomal formulation significantly reduced the toxicity of free oxaliplatin, as evidenced by the increased median survival time of the rats. These findings suggest that liposomal encapsulation of oxaliplatin may help mitigate the adverse effects associated with the free drug and improve the overall safety profile. These studies underscore the potential of liposomal drug delivery systems for brain tumor therapy. By incorporating specific ligands or functional molecules onto liposomes, such as TRAIL, or by encapsulating chemotherapeutic drugs, such as oxaliplatin, within liposomes, targeted drug delivery and improved therapeutic outcomes can be achieved. Further research is needed to optimize these liposomal formulations, including investigating their long-term safety, evaluating their efficacy in larger animal models or clinical trials, and exploring potential combination strategies to enhance their anti-tumor effects.

Kong et al. [144] conducted another study where they incorporated the natural chemical resveratrol into the lipid bilayer of epirubicin-encapsulating liposomes. The liposomes were further modified with p-aminophenyl-α-D-manno-pyranoside (MAN) and wheat germ agglutinin (WGA) on their surface. The multifunctional liposomes were tested *in vitro* on glioma cells and a BBB model and *in vivo* in C6 glioma-bearing rats. The results demonstrated high drug uptake (~70.5%) for epirubicin-WAN-EGA-resveratrol liposomes, highlighting their strong targeting potential compared to control liposomes, which exhibited a drug uptake value of 11%. The liposomes exhibited a significantly higher drug uptake value compared to control liposomes, demonstrating their enhanced ability to target glioma cells. This approach shows the potential for combining multiple targeting strategies to improve the specificity and efficacy of liposomal drug delivery systems in brain cancer treatment.

To target CD13, a transmembrane protein overexpressed in glioma cells, Zhao et al. [145] developed liposomes and enhanced their penetration using focused transcranial ultrasound. The liposomes had a diameter of 157.9 ± 58.33 nm, and the transfection efficacy was 20%. The researchers reported an approximately 8.5-fold increase in shRNA delivery to gliomas in rats, improving survival outcomes. The liposomes exhibited a relatively small diameter and demonstrated efficient shRNA delivery to gliomas in rats, resulting in improved survival outcomes. This approach highlights the importance of combining targeted ligands with physical methods, such as ultrasound, to enhance liposomal formulations’ delivery and therapeutic effects. Therefore, functionalization with specific ligands, encapsulation of chemotherapeutic agents, and combination therapies all contribute to improved tumor targeting, increased drug concentrations at the tumor site, and enhanced therapeutic outcomes. These advancements hold promise for developing more effective treatments for glioblastoma and other brain cancers. These studies collectively emphasize the significance of Functionalization, encapsulation, and combination therapies in liposomal drug delivery for brain tumor treatment. The use of specific ligands, such as MAN, WGA, or CD13-targeting agents, enhances the targeting potential of liposomes, leading to increased drug concentrations at the tumor site and improved therapeutic efficacy. Furthermore, encapsulating chemotherapeutic agents within liposomes allows for controlled release and protection of the drug, minimizing systemic toxicity while maximizing the therapeutic effect. Further research is warranted to optimize the design and formulation of functionalized liposomes, assess their long-term safety and efficacy in larger animal models or clinical trials, and explore their potential for combination therapies with other treatment modalities. By overcoming the challenges associated with brain tumor treatment, functionalized liposomes have the potential to revolutionize the field and improve patient outcomes.

In another study conducted by Song et al. [146], glucose-functionalized liposomes (gLTP) co-loaded with temozolomide (TMZ) and a pro-apoptotic peptide (PAP) demonstrated the ability to penetrate the BBB and release the encapsulated TMZ and PAP through the glucose-GLUT1 pathway. The PAP damages mitochondria and reduces ATP production, making GBM cells more susceptible to TMZ-mediated treatment. Functionalization with glucose allowed the liposomes to utilize the glucose transporter 1 (GLUT1) pathway, which is highly expressed on the surface of GBM cells. This specific targeting mechanism enhances the uptake of the liposomes and facilitates the release of TMZ and PAP within the cancer cells. The combination of PAP-induced mitochondrial damage and TMZ-mediated treatment synergistically increases the susceptibility of GBM cells to the therapy. This approach demonstrates the potential of utilizing functionalized liposomes to overcome the challenges associated with the BBB and achieve targeted drug delivery to GBM.

Liu et al. [147] used a reverse sequence of RGD (dGR) that was linked to a cell-penetrating peptide (CPP) (octa-arginine) to generate a CendR (R/KXXR/K) peptide. This tandem peptide, R8-dGR (RRRRRRRRdGR), binds to both integrin v3 and neuropilin-1 receptors. Paclitaxel was loaded into these functionalized liposomes, designated as PTX-R8-dGR-Lip. The functionalization with R8-dGR allowed the liposomes to bind to both integrin v3 and neuropilin-1 receptors, which are overexpressed on tumor cells. Thus, the liposomes prevented the formation of vasculogenic mimicry channels and exhibited the most significant anti-proliferation impact on both tumor cells and cancer stem cells *in vitro*. The presented dual-targeting approach enhances the internalization of the liposomes into the cancer cells and improves the delivery of paclitaxel. The PTX-R8-dGR-Lip liposomes exhibited significant anti-proliferation effects on both tumor cells and cancer stem cells *in vitro.* This strategy highlights the potential of using functionalized liposomes to simultaneously target multiple receptors and improve anti-cancer drugs’ therapeutic efficacy. These studies demonstrate the importance of functionalizing liposomes to achieve targeted drug delivery and enhance the therapeutic effects in the treatment of GBM and other cancers. By utilizing specific ligands or peptides that can bind to receptors overexpressed on cancer cells, functionalized liposomes can selectively deliver drugs to the tumor site, minimizing off-target effects and improving treatment outcomes. Further research is needed to optimize the design and formulation of functionalized liposomes, evaluate their safety and efficacy in preclinical and clinical settings, and explore their potential for combination therapies with other treatment modalities. With continued advancements in functionalized liposomes, personalized and targeted treatments for GBM and other challenging cancers can be developed, potentially improving patient outcomes and quality of life.

In another study of a CPP, Lakkadwala et al. [148] developed a dual-functionalized liposome delivery system by conjugating a cell-penetrating peptide (CPP) to Tf-liposomes (Tf-Pen-conjugated liposomes). The platform aimed to enhance the delivery of the anti-cancer chemotherapeutic drug 5-fluorouracil (5-FU) across the BBB and into tumor cells, cellular uptake studies of glioblastoma (U87) cells using these dual-functionalized liposomes demonstrated significantly greater cellular uptake compared to other formulations. Furthermore, the formulation exhibited considerably higher levels of apoptosis *in vitro*. The treatment also resulted in significant tumor shrinkage and demonstrated excellent blood compatibility. Overall, the findings of this study highlight the potential of the dual-functionalized liposome delivery system as a viable strategy for brain tumor therapy. By combining the advantages of CPP-mediated cellular uptake and Tf-mediated targeting, these liposomes can improve drug delivery across the BBB and enhance the therapeutic outcomes for patients with glioblastoma. Further research is necessary to evaluate the performance of the dual-functionalized liposomes in preclinical and clinical settings, assess their long-term safety, and optimize their formulation for maximum efficacy. Nonetheless, this study contributes to developing innovative strategies for targeted drug delivery in brain tumor therapy, offering hope for improved treatment options for patients with glioblastoma. Table 3 summarizes the aforementioned preclinical studies.

### 3.2. Clinical Trials

Unfortunately, the therapeutic application of liposomes for targeted drug delivery to the brain is still in its early stages of development. However, several licensed liposomal medications have been approved for clinical use, and ongoing clinical trials are investigating their potential.

In a phase 1 trial conducted by Lippens et al., 14 children diagnosed with gliomas were treated with the liposomal compound DaunoXome. The study reported positive responses in six children, with two achieving full responses, one relapsing after 3 months, and three showing partial responses. Two children had stable disease, while six experienced progressive tumor growth [149]. While positive responses were observed in some children, including complete and partial responses, there were also cases of relapse, stable disease, and progressive tumor growth. These outcomes demonstrate the heterogeneity of treatment responses and the need for further investigation to identify factors that contribute to treatment success or failure.

Wagner et al. conducted a trial involving eight children with high-grade malignant brain tumors treated with Doxil^®^ and oral topotecan. Among the subjects, four showed stable disease, while four experienced progressive disease [150]. These results highlight the challenges in achieving consistent and favorable treatment outcomes using liposomal therapies in pediatric brain tumors. A study by Marina et al. examined a cohort of 22 children with refractory brain tumors. The results showed that only 3 out of the 21 patients had a stable response to the treatment, while the remaining 18 experienced a worsening of their condition [151]. These results highlight the challenges in achieving consistent and favorable treatment outcomes using liposomal therapies in pediatric brain tumors. Only a small fraction of patients experienced a stable response, while the majority saw a worsening of their condition. This underscores the complexity of treating refractory brain tumors and the need for more effective treatment strategies.

Another study conducted by Brenner et al. [152] explored the safety and feasibility of using 186RNL nanoliposomes in combination with radiotherapy for recurrent gliomas in 18 patients. The study demonstrated that after receiving the RNL treatment, six patients were still alive. The fact that 6 out of 18 patients were still alive after receiving the RNL treatment is an encouraging outcome. It suggests that the combination of nanoliposomes and radiotherapy may have a positive impact on patient survival. The study’s findings demonstrate the potential of liposomal drug delivery systems to enhance the efficacy of existing treatments for recurrent gliomas. By encapsulating the therapeutic agents within nanoliposomes, targeted delivery to the tumor site can be achieved, potentially increasing the drug’s effectiveness while minimizing off-target side effects. The combination with radiotherapy further enhances the treatment’s impact by leveraging the synergistic effects of both modalities. However, it is important to acknowledge the limitations, including the small sample size and the need for further research to validate the results. Larger clinical trials are necessary to establish the safety, efficacy, and long-term outcomes of using 186RNL nanoliposomes in combination with radiotherapy for recurrent gliomas.

In a phase 2 trial, Ananda et al. investigated the effects of Doxil^®^ and Temozolomide for the treatment of GBM among 40 patients. The median progression-free survival (PFS) was 13.4 months, while the median time to progression was 6.2 months. One patient demonstrated a full response, twenty-eight had stable disease, and five showed disease progression [153]. The stable disease observed in a significant number of patients indicates a potential benefit of this liposomal therapy in controlling tumor growth. However, it is important to consider that the study had a relatively small sample size, and further research is necessary to confirm these findings and assess overall survival rates. These studies contribute to our understanding of the effectiveness and safety of liposomal therapies in the treatment of brain tumors. While the results are mixed, with some patients demonstrating positive responses and prolonged survival, there is still a need for larger clinical trials to establish the efficacy and optimal use of liposomal formulations. Furthermore, it is crucial to continue refining and developing novel liposomal formulations, exploring combination therapies, and investigating the underlying mechanisms of drug resistance to improve treatment outcomes for patients with brain tumors. Further research is necessary to optimize liposomal formulations, improve drug delivery strategies, and better understand the underlying mechanisms of drug resistance and treatment response in brain tumors. It is crucial to continue exploring new approaches and combination therapies to enhance the efficacy of liposomal drug delivery systems and improve outcomes for patients with brain tumors. Table 4 presents a summary of clinical studies of liposomal drug delivery to the brain.

## 4. Concluding Remarks

This review has presented an overview of the application of liposomes for the targeted treatment of brain tumors. Numerous studies have demonstrated significant tumor reduction with minimal side effects using liposomal-based approaches, both *in vitro* and *in vivo*. Liposomes possess several advantageous characteristics that make them versatile and adaptable nanocarriers for drug delivery to the brain. They can encapsulate both hydrophobic and hydrophilic drugs, are biodegradable and biocompatible, and have the ability to accumulate in tumor cells. Furthermore, liposomes enhance the stability of chemotherapeutic drugs, reduce toxicity, and increase efficacy. Through the incorporation of site-specific ligands, liposomes enable the active targeting of tissues, resulting in localized drug release and minimizing the adverse effects on healthy tissues. Additionally, liposomes, due to their resemblance to cell membranes, can efficiently permeate biological membranes, thereby enhancing the effectiveness and therapeutic benefits of the drug.

However, liposomes also face some limitations. The BBB poses a significant challenge to drug delivery to the brain, but liposomal formulations have demonstrated effectiveness in overcoming this obstacle through various BBB-facilitated transport mechanisms. Several challenges still impede the translation of liposomal-based therapies for brain tumors from the laboratory to clinical practice. Liposomes have a few drawbacks related to the physical and chemical stability of these nanoparticles. Physical instability can occur due to temperature changes, freezing and thawing cycles, and mechanical stress during storage or handling. Chemical degradation, such as lipid oxidation or hydrolysis, can compromise their stability. To achieve long-term stability of liposomes, several techniques can be employed. Lyophilization and cryopreservation help minimize degradation by removing water or freezing liposomes at ultra-low temperatures, respectively. Incorporating stabilizing agents such as cholesterol, surfactants, or polymers prevents aggregation and reduces leakage. Choosing lipids with high stability and incorporating antioxidants protects liposomes from degradation. Other challenges include biosafety assessments by health regulatory authorities, biological obstacles related to liposome administration in humans, commercial-scale production, and the associated high costs. Currently, there is a lack of established criteria for determining the safety of nanodrugs, including liposomes. Traditional methods used for evaluating the safety of conventional medications may not accurately assess the safety of liposomes. Changes in physicochemical properties such as size, shape, surface area, and aggregation at larger scales can impact biodistribution and interactions with cells and biomolecules, further complicating safety assessments. Moreover, modifications in the synthetic route, reagents, manufacturing process, or route of administration can affect the toxicity profile and require the re-evaluation of the drug’s safety.

For liposomes to be used in human patients, they need to be synthesized on a large scale with consistent reproducibility. Many liposomal formulations fail to reach the market due to difficulties in scaling up production or challenges in reproducibility. The complex nature of liposomes adds to the challenges of large-scale manufacturing. Liposomes have typically been produced in limited quantities for preclinical and clinical trials, where managing and optimizing the formulation is easier with small batches. However, even slight variations in the manufacturing process during large-scale production can lead to significant changes in physicochemical properties, drug content, size, surface charge, and therapeutic outcomes. Additionally, the high costs associated with scaling up liposome production can hinder the widespread adoption of innovative liposomal formulations. The lack of regulations and standards for liposome manufacturing methods, quality control, and safety and efficacy evaluation further hinder their development and clinical translation. Currently, there is a lack of universally accepted regulatory criteria for the use of liposomes in clinical practice.

In summary, the aforementioned challenges make the transition of liposomes from the laboratory to the clinic expensive and time-consuming. The need for comprehensive safety assessments, large-scale production capabilities, and regulatory standards contribute to the complexity and costliness of bringing liposomal formulations to the market. While the use of liposomal formulations to treat brain tumors is still in its beginning stages, we are optimistic that this promising drug delivery mechanism can portray great success in clinical translation and can significantly improve the poor prognosis of a multitude of brain tumors in patients. With many nanocarrier-based treatments currently undergoing the first levels of clinical trials, many of which aim to treat cancers of the brain, it is hopefully expected to see liposome-based treatment regimens for brain cancer soon.

## Figures and Tables

**Table 2:** Summary of other nanoparticle-based drug delivery systems.

Nanocarrier	Description	Characteristics	Advantages	Disadvantages	Refs.
Carbon nanotubes 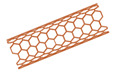	Nanoparticles with a high surface-to-volume ratio, high tensile strength and stiffness, and high drug-loading capacity. Encapsulation of drugs is achieved through the utilization of the van der Waals force. While carbon nanotubes are generally not biocompatible, they can be rendered biocompatible by coating them with molecules such as proteins and organic polymers.	-Repeating hexagonal units of sp2 hybridized carbon atoms -Can be single- or double-walled -Passive and endocytosis-independent cellular uptake	-Protects entrapped drug and allows prolonged release -Bioactive moieties can be conjugated -Large internal surface area allows for high encapsulation efficiencies	-Low pK value -Water-insoluble -Biocompatible if functionalized with molecules such as proteins (antibodies, DNA, RNA) or organic polymers	[80]
Gold nanoparticles 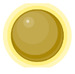	Metal and metal oxide nanoparticles can adopt various shapes, including nanoparticles, nanorods, nanocapsules, nanocuboids, and nanowires. In the context of targeted drug delivery, chemotherapeutic drugs can be chemically attached to the surface of metal nanoparticles or physically loaded into hollow gold or silver nanoparticles. Additionally, their surfaces can be easily functionalized to target specific ligands, further enhancing their potential in drug delivery applications.	-Colloidal gold in aqueous media -Size ranges from 1 to 100 nm	-Facile synthesis protocols -Allows for dual/multiple functionalizations with moieties -Controllable size distribution	-Non-biodegradable -Expensive	[81,82]
QDs 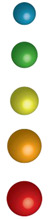	QDs are nanosized semiconductors with unique optical and electrical characteristics. They exhibit bright and narrow-band fluorescence, and their emission can be tailored based on their size and chemistry. Unlike conventional organic label dyes, which have limitations in emitting in the near-infrared spectrum (>650 nm). They can be encapsulated within layers of polymeric or therapeutic material. For instance, a polymer layer can be used to encapsulate hydrophobic drugs, while the surface of the polymer shell can be functionalized with targeting ligands.	-Exhibit distinct optical and electrical features, such as intense and brilliant fluorescence -Can be excited by UV light; when excited electrons return to lower energy states, the energy difference is released as light	-Imaging characteristics -Theranostic applications -Controllable size distribution	-Toxicity/instability limitations -Sensitivity to air -Tendency to form aggregates	[83]
Polymeric nanoparticles 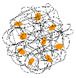	Polymeric nanoparticles are solid colloidal systems in which a pharmacological drug is dissolved, entrapped, encapsulated, or adsorbed onto the polymer matrix. The resulting polymeric nanoparticles can have various structures, ranging from nanospheres to nanocapsules, depending on the method of nanoparticle synthesis. Nanocapsules consist of an oily core where the drug is typically dissolved, surrounded by a polymeric shell that controls the release of the drug from the core. On the other hand, nanospheres are composed of a continuous polymeric network that either dissolves drugs within it or allows drugs to be adsorbed onto its surface. These nanoparticles can be designed to respond to stimuli such as temperature, pH, and redox reactions.	-Particle size ranges from 1 to 1000 nm -May be loaded with active drugs that are either entrapped inside or surface-adsorbed onto the polymeric core -Large NPs used for near-infrared fluorescent *in vivo* imaging	-Tailored drug release profiles -Flexibility in delivery methods -Tendency to accumulate in tumors by EPR -Excellent stability	-Prone to agglomeration -Toxicity in the case of non-degradable polymer usage	[84]
Dendrimers 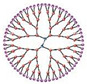	Dendrimers are nanosized, radially symmetric, often polymeric, monodisperse molecules. These molecules branch out from a central molecule, resulting in a highly structured architecture. A first-generation dendrimer forms when the core molecule reacts with monomer molecules having one reactive and two inactive groups. The newly formed periphery of the molecule is then activated, allowing for repetitive branching growth in subsequent layers.	-Nanosized dendrimers have a core, an inner shell, and an outer shell that is symmetrical around the center -Structure is well-defined, homogenous, and monodisperse	-High physiochemical stability due to water solubility, specific molecular weight, and polyvalency -High biocompatibility -Low toxicity	-Batch-to-batch fluctuation in solubilized drug concentration	[85]

**Table 3:** Summary of *in vivo* preclinical studies of liposomal drug delivery to the brain.

Entrance Mechanism	Liposome Type	Payload	Liposome Size	Drug Encapsulation Efficiency	Overview	Reference
Not mentioned	Liposomal Doxorubicin	Doxorubicin	Not mentioned	Not mentioned	In 9 L rat glioma tumors, three weekly FUS and DOX treatments were evaluated. FUS + DOX (N = 8) substantially enhanced median survival time (*p* 0.001) compared to just DOX (N = 6), FUS solo (N = 8), or no therapy (N = 7). FUS + DOX doubled median survival compared to untreated controls, but DOX alone barely doubled it. FUS-only animals did not improve.	[144]
	Liposomal temozolomide formulation (TMZ-lipo)	Temozolomide	148.13 ± 2.66 nm	53%	TMZ-liposomes showed greater C6 tumor-cell-killing efficacy *in vivo* when paired with ultrasonic (US) irradiation, compared to free TMZ as a control Survival time increased from 40 days to 120 days in mice administered with TMZ-lipo + US	[146]
Receptor-Mediated Endocytosis	PEGylated Liposomes	Doxorubicin and Carboplatin (CB)	212 nm ± 10 nm	83.9%	PEG-Lip nanoparticles containing Doxorubicin (DOX) and carboplatin were studied *in vivo* for brain cancer cells Animal survival was 23.1% higher with PEG-Lip-DOX/CB than with DOX + CB.	[136]
	Liposomes	Cell-penetrating peptides (CPPs) and Transferrin (Tf)	155 nm	87.4 ± 3.85%	*In vivo* studies showed 7.7% higher accumulation of brain tumors than the control of liposomes in brain tumors when compared to control.	[145]
	RI7217 (mouse transferrin) and Muscone-Conjugated Liposomes	Docetaxel (DTX)	159.1 ± 4.4 nm	65.37 ± 0.78%	Muscone and RI7217 co-modified DTX liposomes boosted absorption in hCMEC/D3 and U87-MG cells *in vitro* Increased tumor spheroid penetration improved brain targeting *in vivo.* The median survival period of the group given R17217-Muscone-DTX liposome was 24 days, or 1.6 times that of the group given saline.	[139]
	RGD-TPGS-theranostic liposomes	Docetaxel and quantum dots (QDs)	175.6 ± 3.2 nm	68.41 ± 3.56%	RGD-TPGS-theranostic liposomes proved to be 6.47- and 6.98-fold more efficacious than DocelTM *in vivo.* RGD-TPGS liposomes successfully decreased ROS production and showed no evidence of brain injury or edema.	[132]
	Thermosensitive magnetic liposomes (TML)	Camptosar (CPT-11)) and DOX coated with magnetic Fe_3_O_4_ nanoparticles and conjugated with Cetuximab (CET)	193.7 ± 2.3 nm	87.9 ± 1.4%	Enhanced cellular uptake *in vivo*. High biocompatibility and significant tumor shrinkage *in vivo* when compared to control. Survival time increased from 22 days to 30 days in mice administered with TML-CPT-11 liposomes and in conjunction with magnetic guidance	[133]
	PEGylated liposomes (Lipoxal)	Oxaliplatin	118.5 nm	54%	*In vivo* studies in F98 murine models showed a higher accumulation of Lipoxal in tumor cells than the free drug administration. Survival time increased from 30 days to 38.5 days in mice administered with Oxaliplatin + LipoxalA, and notable increases in median survival times were observed.	[140]
Receptor-Mediated Transcytosis	Glutathione doxorubicin-PEGylated liposome (2B3-101)	Doxil^®^/Caelyx^®^	95 nm, P.D < 0.1%	>90%	*In vitro* studies showed complete tumor regression in 3 out of 9 rats. Ten-fold DOX accumulation was noted in animals treated with 2B3-101 when compared to free Doxil^®^/Caelyx^®^.	[146]
	Glutathione doxorubicin-PEGylated liposome (2B3-101)	Doxil^®^/Caelyx^®^	97 nm	Not mentioned	*In vitro* studies showed that four-fold DOX accumulation was noted in animals treated with 2B3-101 when compared to free Doxil^®^/Caelyx^®^.	[147]
	Glucose-functionalized liposomes (gLTP)	Temozolomide (TMZ) and pro-apoptotic peptide (PAP)	133 nm	79.32%	*In vivo* studies showed higher susceptibility of GBM cells than controls. Median survival time increased from 10 days to 30 days.	[148]

**Table 4:** Summary of completed clinical studies of liposomal drug delivery to the brain.

Compound	Lipid Composition	Payload	Trial Phase	Indications	Ref.
DaunoXome	DSPC, Cholesterol	Daunorubicin	I	Pediatric Gliomas	[149]
Doxil^®^/Caelyx	HSPC, Cholesterol, DSPE-PEG_2000_	DOX	II	GBM, Pediatric Gliomas	[153]
Doxil^®^	HSPC, Cholesterol, DSPE-PEG_2000_	DOX	I	Pediatric Glioma	[150]
Doxil^®^	HSPC, Cholesterol, DSPE-PEG_2000_	DOX	I	Refractory brain tumors	[151]
186RNL	Not mentioned	Rhenium-186 Nanoliposome	I	Recurrent GBM	[152]

## Data Availability

Not applicable.

## Abbreviation

BBB	Blood–Brain Barrier
CNS	Central Nervous System
Nanoparticle DDs	Smart Drug Delivery Systems
CDDs	Conventional Drug Delivery Systems
MOA	Mechanism Of Action
TAA	Tumor-Associated Antigen
TSA	Tumor-Specific Antigen
DC	Dendritic Cell
TTP	Time To Progression
OS	Overall Survival
ACT	Adoptive Cell Transfer
IL7	Interleukin-7
IL15	Interleukin-15
CAR	Chimeric Antigen Receptor
GBM	Glioblastoma Multiforme
IL13Rα2	Interleukin-13 Receptor A2
mAb/moAb	Monoclonal Antibody
ADCC	Antibody-Dependent Cellular Cytotoxicity
CMC	Complement-Mediated Cytotoxicity
EGFR	Epidermal Growth Factor Receptor
CTLA4	Cytotoxic T Lymphocyte-Associated Antigen 4
VEGF	Vascular Endothelial Growth Factor
BEV	Bevacizumab
KPS	Karnofsky Performance Status Karnofsky Performance Status
TGF	Transforming Growth Factor
TJ	Tight Junction
AJ	Adherens Junction
ABC	Atp-Binding Cassette
Pgp	P-Glycoprotein
MRP4	Multidrug Resistance Proteins 4
BCRP	Breast Cancer Resistance Protein
DOX	Doxorubicin
BTB	Blood–Tumor Barrier
TMT	Transporter-Mediated Transcytosis
RME	Receptor-Mediated Endocytosis
AMT	Adsorptive-Mediated Transcytosis
RMT	Receptor-Mediated Transcytosis
AMT	Adsorptive- Mediated Transcytosis
ADR.	Adriamycin
LRP	Low-Density Lipoprotein Receptor-Related Proteins
PEG	Polyethylene Glycol
DSPC	Distearoylphosphatidylcholine
HSPC	Hydrogenated soybean phosphatidylcholine
DSPE-PEG2000	1,2-distearoyl-sn-glycero-3-phosphoethanolamine-N-[carboxy(polyethylene glycol)-1000]
HIR	Human Insulin Receptor
RES	Reticuloendothelial System
MOF	Metal–Organic Framework
QD	Quantum Dots
NP	Polymeric Nanoparticles
SUV	Small Unilamellar Vesicles
LUV	Large Unilamellar Vesicles
MLV	Multilamellar Vesicles
FUS	Focused Ultrasound
CB	Carboplatin
CPP	Cell-Penetrating Peptide
Tf	Transferrin
TMZ	Temozolomide
PAP	Pro-Apoptotic Peptide
ELE	Elemene
CTX	Cabazitaxel
TPGS	Alpha-Tocopheryl Polyethylene Glycol 1000 Succinate
TML	Thermosensitive Magnetic Liposome
TRAIL	Tumor Necrosis Factor-Related Apoptosis-Inducing Ligand
MAN	P-Aminophenyl-A-D-Manno-Pyranoside
WGA	Wheat Germ Agglutinin
